# COVID-19 sequelae among competitive athletes: a systematic review: Valerio Flavio Corona

**DOI:** 10.1093/eurpub/ckac131.403

**Published:** 2022-10-25

**Authors:** VF Corona, MR Gualano, A Valz Gris, MR Rossi, L Regazzi, W Ricciardi

**Affiliations:** 1 Department of Life Sciences and Public Health, Catholic University of the Sacred Heart, Rome, Italy; 2 Department of Public Health Sciences, University of Turin, Turin, Italy; 3 Center for Leadership in Medicine Research, Catholic University of the Sacred Heart, Rome, Italy

## Abstract

**Background:**

During the COVID-19 pandemic, several professional athletes from different sports were infected by SARS-CoV-2. The aim of this systematic review was to evaluate the currently available scientific evidence regarding the cardiological, pulmonary, psychological, and combined sequelae, in professional athletes.

**Methods:**

The present systematic review was performed following the PRISMA statements, thereby searching on 3 databases: PubMed, ISI Web of Science, Scopus. Primary studies published between January 2020 and March 2022, investigating symptomatic and instrumental sequelae in competitive athletes after COVID-19 infection, were included.

**Results:**

A total of 1,957 articles were screened, finally 18 were included (6 cohort studies, 2 case-control studies and 10 cross-sectional studies). Studies’ sample size ranged from a minimum of 12 to 1908 athletes playing different sports. In addition, the studies examined the following type of sequelae: 12 cardiological, 2 psychological, 1 pulmonary and 3 combined. Regarding the cardiological field, the prevalence of anomalies in instrumental examinations ranged 0-27.89% for first level tests (echocardiography, electrocardiogram, troponin), and 0-6.21% for second level tests (cardiac magnetic resonance). The prevalence of myocarditis and pericarditis in the athletes ranged from 0 to 3.33%, whereby the prevalence of myocarditis was in the range 0-2.32% and that of pericarditis in the range 0-2.22%.

**Conclusions:**

The results show that post SARS-CoV-2 infection cardiac sequelae have a quite low prevalence among competitive athletes included in our review, but it would be important to set up a gradual and continuous testing approach to preserve sports performance. Public health framework, such as vaccination campaign, is important both at European and international level in order to address potential consequences of infectious diseases among competitive athletes.

**Key messages:**

• It is important to monitor all COVID-19 sequelae in European competitive athletes playing different sports.

• Considering anti-COVID-19 vaccination in competitive athletes as an important preventive measure, to limit the circulation of the virus and the physical consequences that may occur.

